# Evolution of Diverse Effective N_2_-Fixing Microsymbionts of Cicer arietinum following Horizontal Transfer of the Mesorhizobium ciceri CC1192 Symbiosis Integrative and Conjugative Element

**DOI:** 10.1128/AEM.02558-20

**Published:** 2021-02-12

**Authors:** Yvette Hill, Elena Colombi, Emma Bonello, Timothy Haskett, Joshua Ramsay, Graham O’Hara, Jason Terpolilli

**Affiliations:** a Centre for Rhizobium Studies, Murdoch University, Perth, Australia; b Curtin Medical School, Curtin University, Perth, Australia; c Curtin Health Innovation Research Institute, Curtin University, Perth, Australia; University of Michigan-Ann Arbor

**Keywords:** nitrogen fixation, rhizobia, horizontal transfer, symbiosis, chickpea, *Mesorhizobium ciceri*

## Abstract

Rhizobia are soil bacteria capable of forming N_2_-fixing symbioses with legumes, with highly effective strains often selected in agriculture as inoculants to maximize symbiotic N_2_ fixation. When rhizobia in the genus *Mesorhizobium* have been introduced with exotic legumes into farming systems, horizontal transfer of symbiosis integrative and conjugative elements (ICEs) from the inoculant strain to soil bacteria has resulted in the evolution of ineffective N_2_-fixing rhizobia that are competitive for nodulation with the target legume. In Australia, Cicer arietinum (chickpea) has been inoculated since the 1970s with Mesorhizobium ciceri symbiovar ciceri CC1192, a highly effective strain from Israel. Although the full genome sequence of this organism is available, little is known about the mobility of its symbiosis genes and the diversity of cultivated C. arietinum-nodulating organisms. Here, we show that the CC1192 genome harbors a 419-kb symbiosis ICE (ICE*Mc*Sym^1192^) and a 648-kb *repABC*-type plasmid (pMC1192) carrying putative *fix* genes. We sequenced the genomes of 11 C. arietinum nodule isolates from a field site exclusively inoculated with CC1192, and we showed that they were diverse unrelated *Mesorhizobium* strains carrying ICE*Mc*Sym^1192^, which indicated that they had acquired the ICE by environmental transfer. No exconjugants harbored pMc1192, and the plasmid was not essential for N_2_ fixation in CC1192. Laboratory conjugation experiments confirmed that ICE*Mc*Sym^1192^ is mobile, integrating site specifically within the 3′ end of one of the four Ser--tRNA genes in the R7ANS recipient genome. Strikingly, all ICE*Mc*Sym^1192^ exconjugants were as efficient as CC1192 at fixing N_2_ with C. arietinum, demonstrating that ICE transfer does not necessarily yield ineffective microsymbionts as observed previously.

**IMPORTANCE** Symbiotic N_2_ fixation is a key component of sustainable agriculture, and in many parts of the world legumes are inoculated with highly efficient strains of rhizobia to maximize fixed N_2_ inputs into farming systems. Symbiosis genes for *Mesorhizobium* spp. are often carried chromosomally within mobile gene clusters called ICEs. In Australia, where all agricultural legumes and their rhizobia are exotic, horizontal transfer of ICEs from inoculant *Mesorhizobium* strains to native rhizobia has led to the evolution of inefficient strains that outcompete the original inoculant, with the potential to render it ineffective. However, the commercial inoculant strain for Cicer arietinum (chickpea), M. ciceri CC1192, has a mobile symbiosis ICE (ICE*Mc*Sym^1192^) that can support high rates of N_2_ fixation following either environmental or laboratory transfer into diverse *Mesorhizobium* backgrounds, demonstrating that ICE transfer does not necessarily yield ineffective microsymbionts as observed previously.

## Abstract (precis)

Symbiotic N_2_ fixation is a key component of sustainable agriculture, and in many parts of the world legumes are inoculated with highly efficient strains of rhizobia to maximize fixed N_2_ inputs into farming systems. Symbiosis genes for *Mesorhizobium* spp. are often carried chromosomally within mobile gene clusters called ICEs.

## INTRODUCTION

Rhizobia are soil bacteria that are capable of forming a symbiotic association with legumes. The symbiosis is established when rhizobia infect legume roots, resulting in the formation of root nodules, where atmospheric N_2_ is “fixed” by rhizobia into organic nitrogen that is incorporated into plant tissues. When legumes are grown in rotation with other crops, legume nitrogen enters the soil following plant senescence and decay or deposition from grazing livestock (1). Legumes and rhizobia are often introduced into agriculture to increase soil fertility and to reduce the use of industrially synthesized nitrogenous fertilizers (2, 3). In Australia, all forage and grain legumes are exotic, having been introduced following European colonization in the late 18th century (4, 5). Prior to this, it appears that Australian soils lacked rhizobia capable of forming effective N_2_-fixing symbioses with introduced legumes (6, 7). Therefore, many inoculant strains have been sourced from other parts of the world through dedicated selection programs to match plant host with rhizobia that are well adapted to these environments and capable of fixing large amounts of N_2_ with the target legume (7–9).

Since the 1990s, it has become increasingly clear that the genetic diversity of resident strains in Australian soils that are capable of nodulating agricultural legumes far exceeds the diversity of strains introduced as inoculants (10–13). For some legumes, these resident or “naturalized” rhizobia are present in such large numbers that they, and not the inoculant strain, dominate as nodule occupants (14, 15). These resident strains often fix N_2_ suboptimally (16–18), posing a significant constraint to maximizing symbiotic N_2_ fixation. While the origin of resident strains has been debated for some time (60), mounting evidence for the role of horizontal gene transfer as a driver for bacterial evolution (19–21) indicates that this is likely a key contributor to their evolution.

Rhizobial symbiosis genes, which include the nodulation genes (*nod*, *noe*, and *nol* [collectively, *nod* genes]) (22) and nitrogen fixation genes (*nif* and *fix*), are critical to the establishment of N_2_-fixing legume associations (23). Symbiosis genes can be carried on plasmids or clustered on the bacterial chromosome, and this arrangement appears to be largely genus specific (24, 25). For rhizobia in the genus *Mesorhizobium*, symbiosis genes are carried chromosomally within integrative and conjugative elements (ICEs), which can be either monopartite or tripartite in structure (26–28).

The first symbiosis ICE discovered was the monopartite ICE*Ml*Sym^R7A^ in Mesorhizobium japonicum (formerly Mesorhizobium loti) R7A (27, 29), following inoculation of the introduced legume Lotus corniculatus in New Zealand. Rhizobia isolated several years later from L. corniculatus root nodules were genetically distinct from the inoculant strain but harbored the R7A symbiosis ICE (30). ICE*Ml*Sym^R7A^ is integrated at the 3′ end of the Phe-tRNA gene in the R7A chromosome, flanked by attachment sites (*attL* and *attR*) that contain identical 17-bp sequences (26). Excision of ICE*Ml*Sym^R7A^ from the chromosome is catalyzed by integrase (IntS)- and recombination directionality factor (RdfS)-mediated site-specific recombination between *attL* and *attR*. The resulting episome can subsequently transfer by conjugation to a recipient cell, integrating by IntS-dependent recombination at the 3′ end of the Phe--tRNA gene (26). Importantly, field experiments at a second site in New Zealand showed over a four-year period that 75% of root nodules sampled contained diverse *Mesorhizobium* strains that had acquired ICE*Ml*Sym^R7A^. This converted them into L. corniculatus-nodulating microsymbionts that outcompeted the inoculant for nodulation of the legume (31).

Novel microsymbionts have also evolved in Australian soils after introduction of the pasture legume Biserrula pelecinus and Mesorhizobium ciceri symbiovar (sv.) biserrulae WSM1271 from the Mediterranean into western Australia (9, 18, 32). Six years after inoculation at a site with no preexisting B. pelecinus-nodulating rhizobia, novel isolates genetically distinct from the inoculant were recovered from B. pelecinus nodules. Crucially, all of the novel isolates were either completely ineffective (i.e., nodulated B. pelecinus but did not fix N_2_) or fixed significantly less N_2_ than did WSM1271 (18). Detailed analysis of two strains (Mesorhizobium australicum WSM2073 and Mesorhizobium opportunistum WSM2075) showed that they both had acquired the WSM1271 tripartite symbiosis ICE (ICE*Mc*Sym^1271^) by horizontal transfer (28). A similar analysis following inoculation of B. pelecinus with M. ciceri sv. biserrulae WSM1497 found that 47.5% of the strains isolated from nodules were novel and all of them were less effective than WSM1497 at fixing N_2_, with six being completely ineffective (18). Therefore, horizontal transfer of symbiosis ICEs from *Mesorhizobium* inoculants has resulted in the evolution of novel strains that are competitive but less effective at fixing N_2_ than the inoculant strain.

Cicer arietinum (chickpea) is the largest legume crop in Australia (33), grown predominantly in northeastern Australia and extending to regions in the southeast as well as parts of western Australia. C. arietinum forms a N_2_-fixing symbiosis with rhizobia in the genus *Mesorhizobium* (34); however, when this grain legume was first introduced in the 1970s, Australian soils appeared to lack compatible C. arietinum-nodulating rhizobia (7). This led to the selection of M. ciceri sv. ciceri strain CC1192 from Israel and its subsequent use as a commercial inoculant for C. arietinum across the country for more than 40 years (7, 15, 35). Despite this, Elias and Herridge (36) reported that 53% of C. arietinum nodules sampled from 26 farms in eastern Australia were occupied by strains genetically distinct from CC1192. Furthermore, 41% of a subset of those strains were significantly less effective than CC1192 at fixing N_2_ with C. arietinum (36).

The presence of soil populations of suboptimally effective C. arietinum-nodulating rhizobia could reduce the benefits to agriculture of symbiotic N_2_ fixation following inoculation with CC1192. Here, we investigate the genome of CC1192 and describe the structure and genetic content of its symbiosis ICE, ICE*Mc*Sym^1192^. We show that genes essential for symbiosis are carried on ICE*Mc*Sym^1192^ and that it is transferable *in vitro* and in the field. Remarkably, transfer of the ICE into genetically diverse *Mesorhizobium* strains yields strains that are effective microsymbionts of C. arietinum, showing that ICE transfer does not always yield inefficient N_2_-fixing strains.

## RESULTS

### Genetically diverse strains of *Mesorhizobium* nodulate C. arietinum.

**FIG 1:**
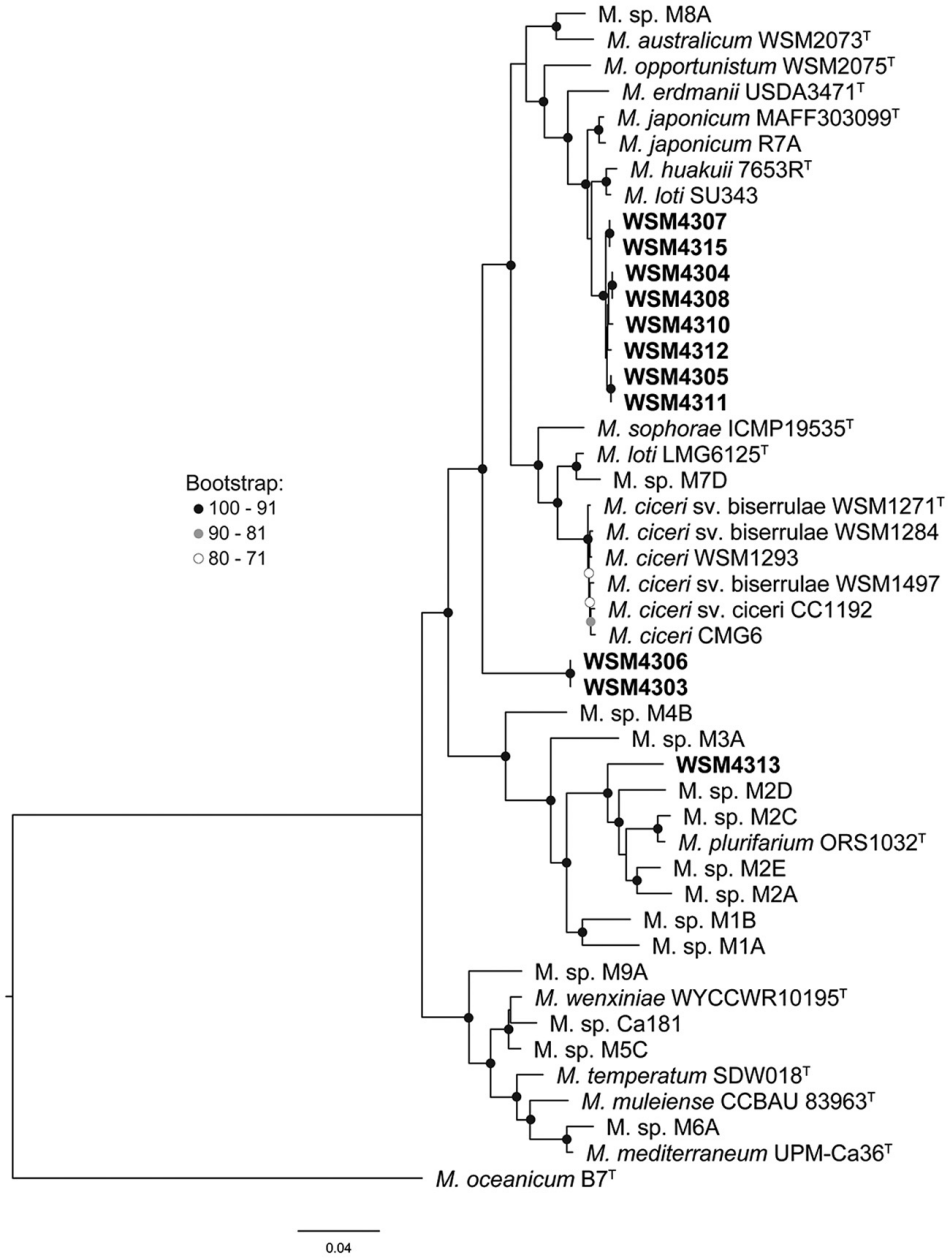
Genome tree of *Mesorhizobium* isolates prepared with bcgTree using a concatenated alignment of 107 core proteins conserved across bacterial genera to compare organisms. The tree was constructed using the RAxML program with 100 bootstraps. In this tree, the genome of M. ciceri Ca181 has been renamed M. sp Ca181 because it is genetically distinct from the M. ciceri clade.

To investigate the genetic diversity of rhizobia isolated from field-cultivated C. arietinum, we performed whole-genome sequencing of 11 strains (WSM4303 to WSM4308, WSM4310 to WSM4313, and WSM4315) available from the work of Elias and Herridge (36). We compared these sequences to those of selected *Mesorhizobium* type strains, commercial inoculants released in Australia, and strains from the recent study of *Cicer*-nodulating rhizobia by Greenlon et al. (37) by constructing a genome tree with bcgTree based on 107 essential single-copy core genes (Fig. 1). The 11 C. arietinum strains grouped into three clades, which were not closely related to the commercial inoculant strains for C. arietinum (M. ciceri sv. ciceri CC1192), Biserrula pelecinus (M. ciceri sv. biserrulae WSM1497 and WSM1271), or *Lotus* spp. (M. loti SU343). The largest clade contained eight of the strains, subdivided into five subgroups (WSM4307 with WSM4315, WSM4304 with WSM4308, WSM4305 with WSM4311, and WSM4310 and WSM4312 each on a separate branch), while WSM4306 and WSM4303 grouped together on a separate branch, only distantly related to other strains in the tree. WSM4313 grouped separately and was most closely related to several strains isolated from C. arietinum growing in Ethiopia (*Mesorhizobium* sp. strains M2D, M2E, and M2A) and Turkey (*Mesorhizobium* sp. strain M2C) and Mesorhizobium plurfarium from Acacia senegal in West Africa.

**FIG 2:**
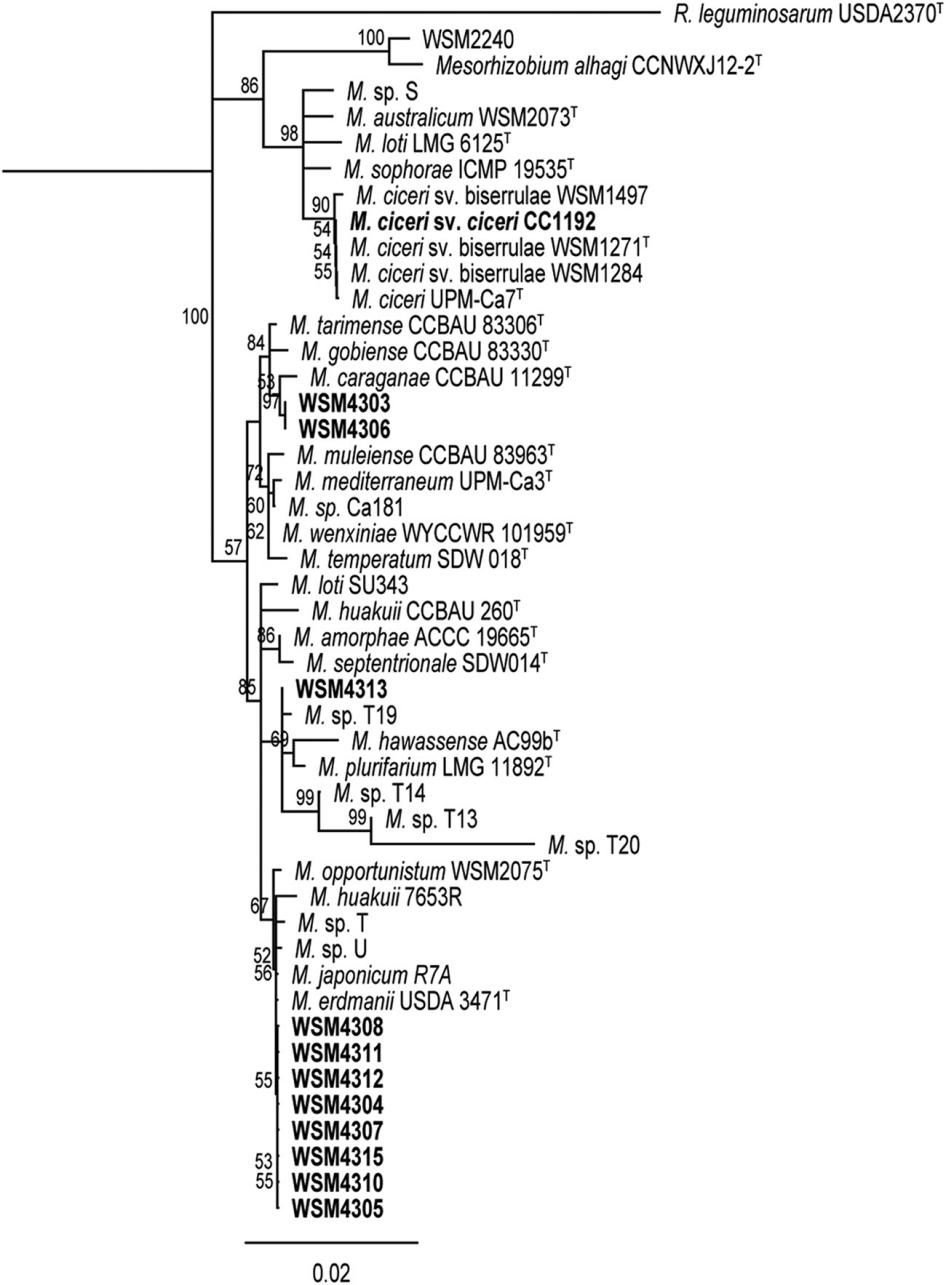
Neighbor-joining phylogenetic tree of 16S rRNA gene sequences of *Mesorhizobium* isolates from this study and other rhizobia obtained from GenBank (type strains are indicated by T in the strain identification). Isolates from this study and the commercial inoculant M. ciceri CC1192 are indicated in bold type. Analyses were conducted in Geneious v11.1.5 using the Tamura-Nei method to determine genetic distances, with Rhizobium leguminosarum bv. *viciae* USDA 2370^T^ as the outgroup. Branch values of <50% from 5,000 bootstraps are not shown.

Whole-genome sequences are not available for *Mesorhizobium* strains isolated from Australian native legumes; therefore, we were unable to extend this core gene analysis to those organisms. Instead, we constructed a 16S rRNA tree using the 11 strains, available sequences from native legume-nodulating *Mesorhizobium* strains, and commercial inoculant and selected type strains from the genome tree (Fig. 2). Consistent with the genome tree, the 11 strains grouped into the same three clades, with the largest clade being clustered with *Mesorhizobium* strains U and T, isolated from the native legumes Acacia obliquinervia and Goodia lotifolia from southeastern Australia (38). Similarly, WSM4313 grouped closely with *Mesorhizobium* grouping T19, representing a genospecies comprising 39 strains isolated from native legumes Acacia stenophylla and Acacia salicina growing in the Murray River Basin area of southeastern Australia (39), while the remaining two strains (WSM4303 and WSM4306) did not group closely with the *Mesorhizobium* strains from Australian native legumes. Therefore, the 11 strains appeared to be novel C. arietinum-nodulating *Mesorhizobium* strains. Given that Australian soils were reported to lack compatible C. arietinum-nodulating rhizobia prior to the release of CC1192 (7), this finding suggested these 11 strains might have acquired their symbiosis genes following introduction of the inoculant strain.

### Environmental and laboratory transfer of ICE*Mc*Sym^1192^ produces effective symbionts.

**FIG 3:**
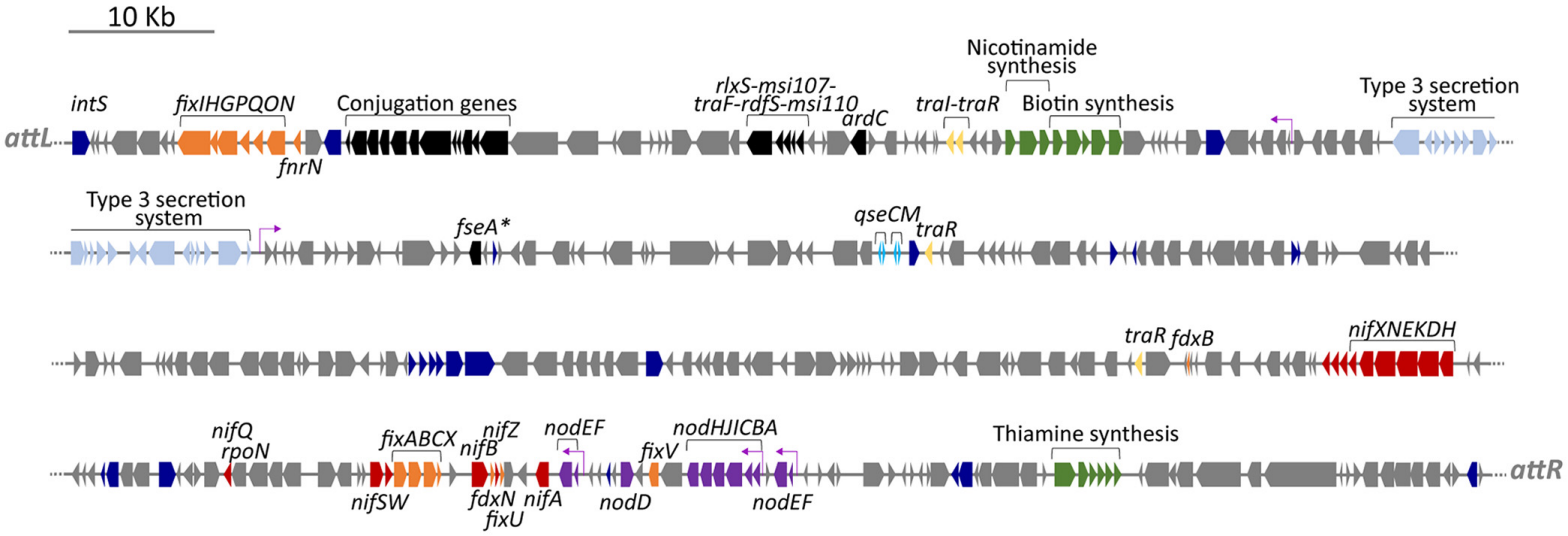
Structure of ICE*Mc*Sym^CC1192^. M. ciceri CC1192 carries a 418,907-bp symbiosis ICE (base pairs 4216431 to 4635338) adjacent to the sole Ser--tRNA (A4R28_RS20655) in the M. ciceri CC1192 genome, with the ICE containing 373 predicted coding sequences. Gene annotations are color coded as follows: blue, mobile genes such as transposases, integrases, and recombinases; pale blue, type 3 secretion systems; black, ICE transfer genes; yellow, quorum-sensing genes; green, vitamin biosynthesis genes; red, nitrogen fixation *nif* genes; orange, nitrogen fixation *fix* genes; purple, nodulation *nod* genes. Putative *nod* boxes are indicated by arrows. An asterisk (*) indicates frameshifting required for gene to be translated. Gene coordinates are provided in Table S1 in the supplemental material.

**FIG 4:**
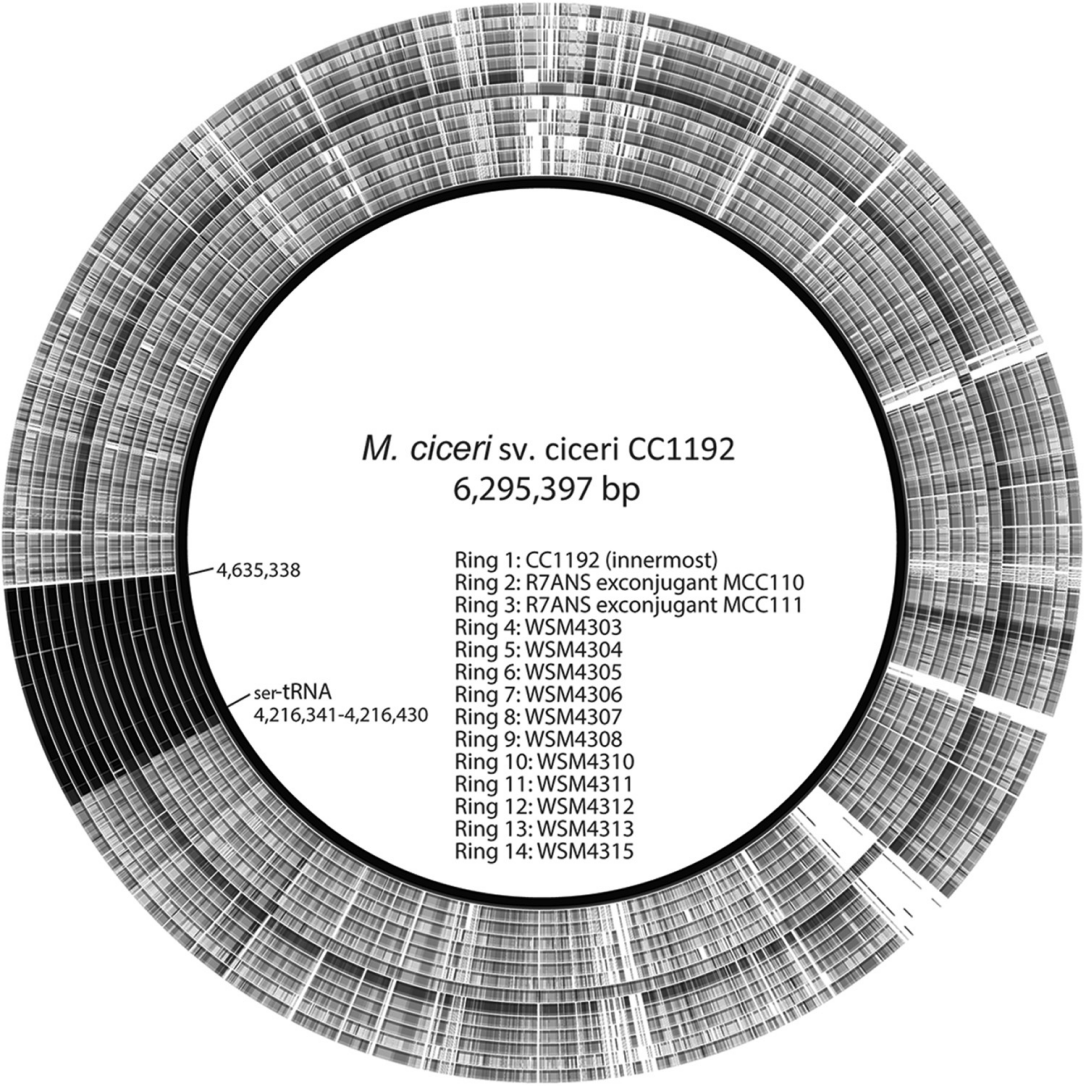
Conservation of ICE*Mc*Sym^1192^ in CC1192 (inner circle), two exconjugants of R7ANS (MCC110 and MCC111), and 11 novel C. arietinum-nodulating field isolates, compared to the CC1192 reference genome. Circular BLASTN alignments of CC1192 with MCC110, MCC111, WSM4303, WSM4304, WSM4305, WSM4306, WSM4307, WSM4308, WSM4310, WSM4311, WSM4312, WSM4313, and WSM4315 were carried out using BRIG (68). Black regions indicate >99% conserved nucleotide identity.

**FIG 5:**
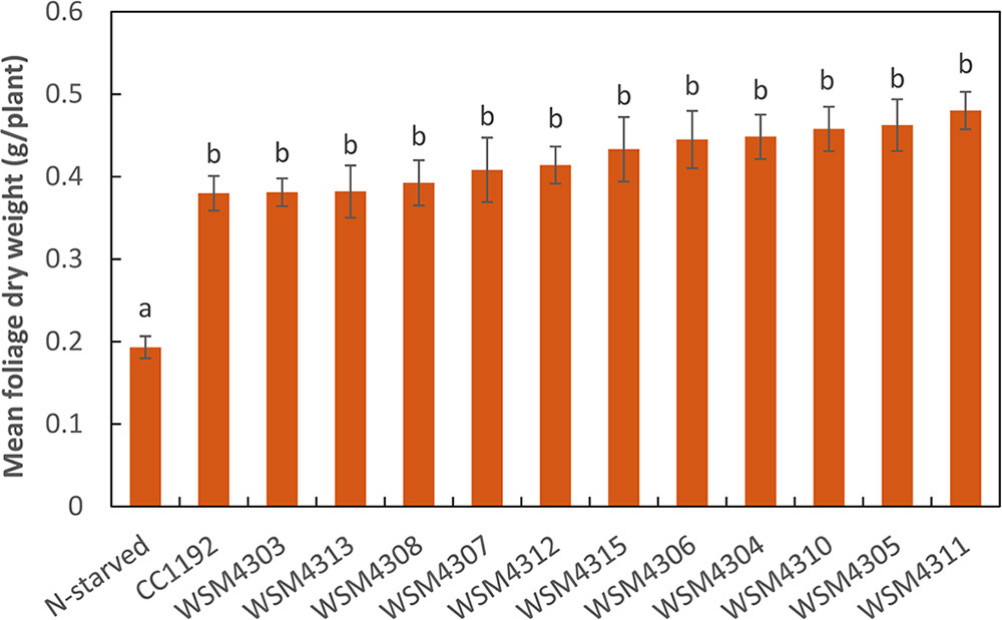
Mean foliage dry weight of Cicer arietinum cv. Neelam inoculated separately with 11 novel isolates and the commercial inoculant M. ciceri CC1192 and grown for 49 days. Treatments are shown with standard errors of the means, and the treaments that share a letter are not significantly different according to the Tukey HSD test (*P ≤ *0.05). Uninoculated control plants received no nitrogen (N-starved) and were devoid of nodules.

Previous work predicted that CC1192 harbored a 419-kb symbiosis ICE within its 6.29-Mbp chromosome (GenBank accession number CP015062) (40), referred to as ICE*Mc*Sym^1192^. ICE*Mc*Sym^1192^ is a monopartite ICE flanked by 20-bp repeat DNA sequences (5′-GAATCCCTCCCTCTCCGCCA-3′) identical to the 3′ end of the Ser--tRNA gene, which presumably contains the core regions of the integrase attachment sites *attL* and *attR* (Fig. 3). The genetic complement of ICE*Mc*Sym^1192^ is broadly similar to that of the well-characterized ICE*Ml*Sym^R7A^ from M. japonicum R7A. ICE*Mc*Sym^1192^ harbors a complete set of core nodulation (*nod*) genes and nitrogen fixation (*nif* and *fix*) genes, as well as an integrase gene (*intS*) distinct from previously characterized symbiosis ICEs (see Table S1 in the supplemental material for gene coordinates), with conjugation genes (*trb* gene cluster and *rlxS*) and ICE excision regulation genes (*rdfS* and *fseA*) (Fig. 3) highly similar to those of ICE*Ml*Sym^R7A^ and the tripartite ICE*Mc*Sym^1271^. These characteristics suggested that ICE*Mc*Sym^1192^ is mobile; therefore, we compared the *de novo* assembled genomes of the novel *Mesorhizobium* strains with the CC1192 genome. The entire 419-kb ICE*Mc*Sym^1192^ region was present in all 11 strains, confirming the environmental transfer of ICE*Mc*Sym^1192^ to these strains (Fig. 4). We next investigated how the *Mesorhizobium* recipients of ICE*Mc*Sym^1192^ performed in controlled glasshouse experiments on C. arietinum. Of the 11 strains tested against CC1192, all produced foliage dry weights that were not significantly different from those with CC1192, with mean values ranging from 0.402 to 0.506 g/plant (Fig. 5). Therefore, these data demonstrate that these novel *Mesorhizobium* strains have acquired ICE*Mc*Sym^1192^ and are fully effective at fixing N_2_ on C. arietinum.

We next tested the mobility of ICE*Mc*Sym^1192^ *in vitro*, by carrying out conjugation experiments using R7ANS (the ICE-cured derivative of M. japonicum R7A) as a recipient (26). The neomycin resistance plasmid pPR3 was introduced into R7ANS to enable selection against donor cells. While R7ANS is auxotrophic for biotin, nicotinate, and thiamine, the genes for the biosynthesis of these vitamins (*bioBFDAZ*, *nadABC* and *thiCOSGED*, respectively) are present on ICE*Mc*Sym^1192^ (Fig. 3). R7ANS exconjugants harboring ICE*Mc*Sym^1192^ were isolated on medium lacking biotin and nicotinate. Vitamin prototrophs were acquired on selection plates, with a transfer frequency of 1.02 × 10^−7^ ± 0.52 × 10^−7^ exconjugants per donor (mean ± standard error of the mean).

As with the CC1192 genome, in which there are multiple Ser--tRNA genes (40), five Ser--tRNA genes are present in the R7ANS genome, which are potential integration sites for ICE*Mc*Sym^1192^, although only one (R7A2020_05665) contains a 20-bp region identical to those present in the *attL* and *attR* sites flanking ICE*Mc*Sym^1192^. This indicated that this Ser--tRNA was a likely integration site for ICE*Mc*Sym^1192^ in the R7ANS genome. PCR screening of 10 exconjugants with primers designed to target integration of ICE*Mc*Sym^1192^ at R7A2020_05665 showed all 10 had integrated at this site. To confirm that mating of CC1192 with R7ANS had resulted in the complete transfer of ICE*Mc*Sym^1192^ into the recipient strain, two exconjugant strains, MCC110 (GenBank accession number JADAMJ000000000) and MCC111 (GenBank accession number JADAMK000000000), were selected for whole-genome sequencing. BLASTN comparison of the *de novo* assembled genomes with wild-type CC1192 confirmed that the 419-kb ICE*Mc*Sym^1192^ had been transferred in its entirety into the R7ANS recipient, integrating at Ser--tRNA (R7A2020_05665) (Fig. 4). Therefore, ICE*Mc*Sym^1192^ is a mobile element able to be acquired by the R7ANS recipient strain.

**FIG 6:**
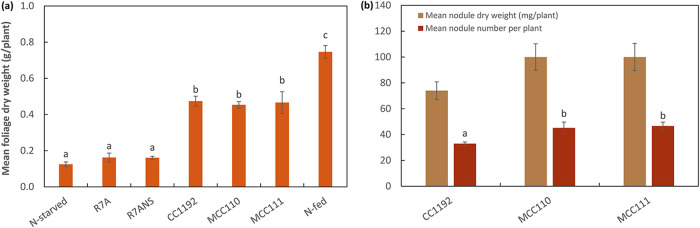
Symbiotic effectiveness of exconjugants of the ICE*Mc*Sym^1192^ mobilized into R7ANS (MCC110 and MCC111), compared to wild-type CC1192, R7A (Mesorhizobium japonicum strain carrying ICE*Mj*Sym^R7A^), and R7ANS (symbiosis ICE-devoid derivative of R7A), on *C. aritetinum* cv. Neelam. (a and b) Mean foliage dry weight (a) and mean dry nodule weight per plant and nodule number of plants (b) grown under nitrogen-limited conditions, inoculated separately with the indicated strains, and grown for 49 days. Uninoculated and N-fed (supplied as KNO_3_) plants were included as negative and positive controls, respectively. Treatments are shown with standard errors of the means, and the treaments that share a letter are not significantly different according to the Tukey HSD test (*P ≤ *0.05). A one-way ANOVA detected no significant difference (*P ≤ *0.05) between the treatments for nodule dry weights.

In previous work with the B. pelecinus-nodulating strain M. ciceri sv. biserrulae WSM1271, laboratory transfer of the WSM1271 tripartite ICE*Mc*Sym^1271^ to R7ANS produced exconjugants that were only partially effective at fixing N_2_ with the legume host (40). To see whether the R7ANS exconjugants that had acquired ICE*Mc*Sym^1192^ similarly showed reduced effectiveness, we examined the symbiotic phenotype of the R7ANS exconjugants MCC110 and MCC111 in comparison with CC1192 on C. arietinum, along with R7ANS and its parent strain M. japonicum R7A. Both R7A and R7ANS failed to nodulate C. arietinum, and plant shoot dry weights were not significantly different from those of uninoculated N-starved controls (Fig. 6a). In contrast, C. arietinum inoculated with MCC110 or MCC111 produced plants with >3.5-fold greater shoot biomass than the N-starved control. These values were not significantly different (*P ≤ *0.05) from the mean shoot dry weights of CC1192-inoculated plants. Although MCC110 and MCC111 produced approximately 23% more nodules (*P < *0.05) than CC1192 on C. arietinum, values for the total nodule mass per plant were not different (*P* = 0.115) (Fig. 6b). Similarly, comparison of nitrogenase activity between wild-type and exconjugant strains by acetylene reduction assays showed that acetylene reduction rates per plant, per nodule, and per unit nodule mass were not significantly different across the three strains (Table 1). Therefore, the transfer of ICE*Mc*Sym^1192^ from CC1192 into R7ANS yields exconjugants that form a symbiosis that is equally effective at fixing N_2_ with C. arietinum, compared with wild-type CC1192.

**TABLE 1:** Acetylene reduction assay of C. arietinum cv. Neelam grown for 49 days and inoculated separately with R7ANS (ICE*Mc*Sym^1192^) exconjugant MCC110, MCC111, or CC1192

Strain	Acetylene reduction^*a*^		
	Per plant (µmol acetylene reduced/plant/h)	Per nodule (nmol acetylene reduced/nodule/h)	Per nodule mass (nmol acetylene reduced/mg nodule/h)
CC1192	1.020 ± 0.255	32.2 ± 9.1	13.4 ± 2.9
MCC110	0.827 ± 0.259	18.8 ± 5.1	7.90 ± 2.0
MCC111	1.146 ± 0.082	25.2 ± 2.6	12.3 ± 2.1

a Treatment means ± standard errors are shown. A one-way ANOVA detected no significant difference (*P ≤ *0.05) between the treatments.

### The 648-bp plasmid pMc1192 is not essential for N_2_ fixation with C. arietinum.

Although acquisition of the symbiosis genes on ICE*Mc*Sym^1192^ is sufficient to support CC1192 levels of N_2_ fixation in the 11 novel strains of *Mesorhizobium* and R7ANS(ICE*Mc*Sym^1192^) exconjugants, further sequence analysis of the CC1192 complete genome showed additional putative symbiosis-related genes on the *repABC*-type plasmid pMc1192 (GenBank accession number CP015063). Among the 645 predicted coding sequences on this 648,231-bp plasmid are copies of *fixNOQP* (A4R28_RS30260, RS30265, RS30270, and RS30275) and *fixGHI* (A4R28_RS30280, RS30285, and RS30290). These copies are in addition to those on located on ICE*Mc*Sym^1192^ (*fixNOQP*, A4R28_RS20715, RS20710, RS20705, and RS20700; *fixGHI*, A4R28_RS20695, RS20690, and RS20685), with which they share a pairwise average nucleotide identity of 88%. The plasmid *fixGHI* gene cluster also includes *fixS* (A4R28_RS30295), a gene that is absent from the ICE copy of *fixGHI* (Fig. 3). Furthermore, pMc1192 also harbors *fixLJ* (A4R28_30350 and A4R28_30355) and *fixK* (A4R28_30370), which are not present on ICE*Mc*Sym^1192^. The *fixLJ* and *fixK* genes are essential for N_2_ fixation in the Sinorhizobium meliloti-*Medicago* symbiosis, with FixLJ acting as a low-O_2_-sensing two-component sensor-regulator system, which in turn controls expression of transcriptional regulators *fixK* and *nifA* (41).

**FIG 7:**
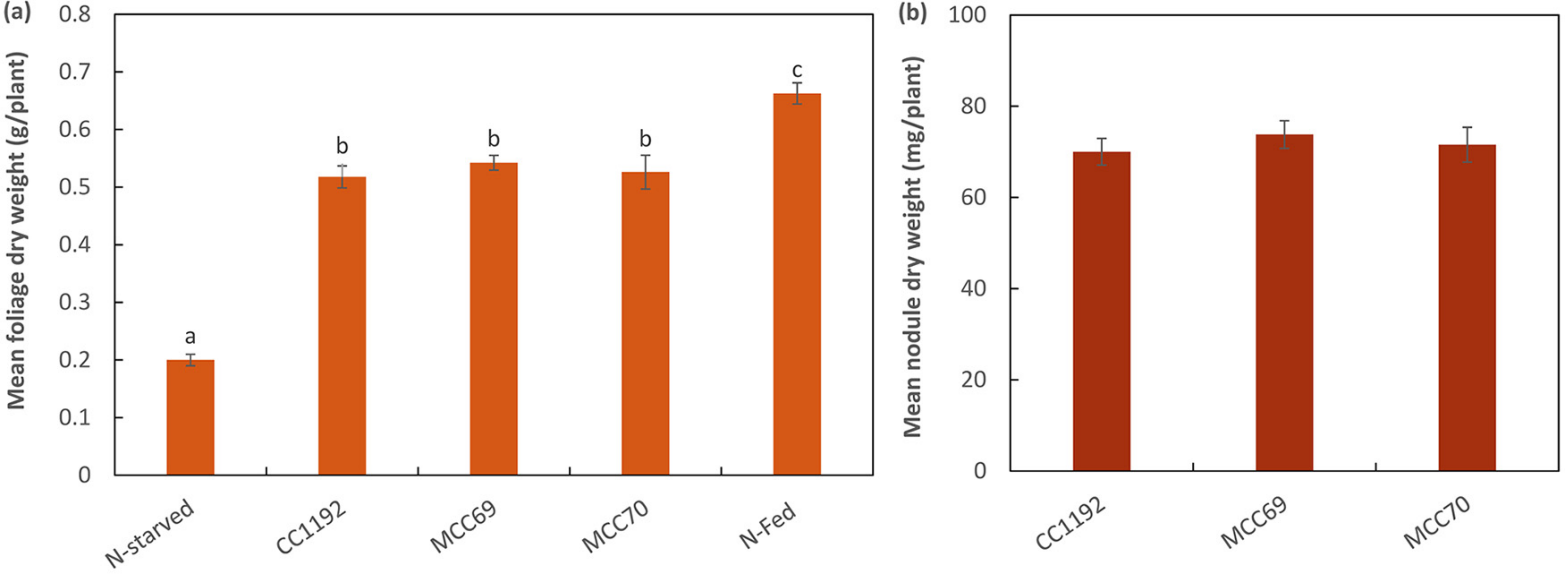
Symbiotic effectiveness of plasmid-cured derivatives of CC1192 (MCC69 and MCC70), compared to CC1192, on C. arietinum cv. Neelam. (a and b) Mean foliage dry weight (a) and mean nodule dry weight (b) per plant of C. arietinum inoculated separately with the indicated strains and grown free of added nitrogen under controlled glasshouse conditions for 44 days. Uninoculated control plants received no nitrogen (N-starved) or nitrogen as KNO_3_ (N-fed), and the roots of these plants were devoid of nodules at harvest. Error bars represent standard errors of the means, and treaments that share a letter are not significantly different, according to the Tukey HSD test (*P ≤ *0.05). A one-way ANOVA detected no significant difference (*P ≤ *0.05) between the treatments for nodule dry weights.

To determine whether the plasmid *fix* genes had a role in the CC1192-C. arietinum symbiosis, plasmid pMc1192 was cured from CC1192 using a plasmid incompatibility approach. Two independently acquired plasmid-cured derivatives of CC1192 (MCC69 and MCC70) were isolated. PCR screening, Eckhardt gel electrophoresis (Fig. S1), and whole-genome sequencing of MCC70 (GenBank accession number SRX9131521) confirmed loss of pMc1192. To determine whether the loss of pMc1192 and the symbiotic genes it contained affected symbiotic performance, the nodulation and N_2_ fixation phenotypes of the two plasmid-cured strains, MCC69 and MCC70, were compared to that of the parent strain CC1192 on C. arietinum (Fig. 7). At 44 days postinoculation, there was no significant difference (*P* ≤ 0.05) among MCC69, MCC70, and CC1192 in either mean shoot dry weight or mean nodule dry weight per plant. Furthermore, pMc1192 was also absent from the genomes of the 11 *Mesorhizobium* strains isolated from field-cultivated C. arietinum, indicating that they had not acquired this plasmid. Therefore, although plasmid pMc1192 harbors predicted *fix* genes, they are not essential to support N_2_ fixation with C. arietinum.

## DISCUSSION

We have demonstrated that Mesorhizobium ciceri sv. ciceri CC1192 harbors a 419-kb monopartite symbiosis ICE, ICE*Mc*Sym^1192^, integrated at one of the four Ser--tRNA genes in the CC1192 genome. The ICE is transferable to the ICE-devoid R7ANS recipient, where it integrates into a 20-bp sequence present at the 3′ end of the Ser--tRNA gene. Furthermore, the identification of ICE*Mc*Sym^1192^ in the genomes of genetically distinct *Mesorhizobium* strains present in C. arietinum nodules is consistent with ICE*Mc*Sym^1192^ transfer in the field. Although Ser--tRNA had been proposed as the integration site for ICE*Mc*Sym^1192^ (40) as well as for 10 other predicted monopartite symbiosis ICEs from *Cicer-*nodulating strains (37), here we have experimentally confirmed this locus as an integration site for symbiosis ICEs. The rate of transfer of ICE*Mc*Sym^1192^ to R7A of 1.05 × 10^−7^ transconjugants per donor is comparable to the ICE*Ml*Sym^R7A^ transfer rate in wild-type M. japonicum R7A (2.5 × 10^−7^ transconjugants per donor) (42) but is more than 1 order of magnitude higher than that for the transfer of ICE*Mc*Sym^1271^ from WSM1271 to R7ANS (4.65 × 10^−8^ transconjugants per donor) (28). The differences in these transfer rates may be related to the monopartite structure of ICE*Mc*Sym^1192^ and ICE*Ml*Sym^R7A^ and their highly similar complements of ICE regulatory genes, compared to the more complex and intricate control system in the tripartite ICE*Mc*Sym^1271^ (43).

Curing CC1192 of plasmid pMc1192 had no impact on the nodulation or N_2_ fixation phenotypes of the resultant strains, and the genomes of the 11 *Mesorhizobium* strains isolated from field-cultivated C. arietinum lacked the plasmid. This indicates that the 645 genes, including *fix* genes (*fixNOPQGHIS* and *fixLJK*), carried on pMc1192 are not essential for symbiosis with C. arietinum. In several rhizobia, FixNOQP and FixGHI are required for production of a high-O_2_-affinity *cbb*_3_-type cytochrome oxidase critical for symbiosis, with mutations in these operons either abolishing or greatly reducing rates of N_2_ fixation (44–47). Given that *fixNOQP* and *fixGHI* are also present on ICE*Mc*Sym^1192^, it is likely that M. ciceri CC1192 relies on these ICE-carried genes to fulfil the symbiotic roles of FixNOQP and FixGHI. Although a definitive role for FixS has yet to be reported for N_2_-fixing rhizobia, *fixS/ccoS* has been shown to be essential for maturation of the microaerobic terminal oxidase *cbb_3_* complex in the photosynthetic purple nonsulfur bacterium Rhodobacter sphaeroides (48, 49). Plasmid pMc1192 appears to harbor the sole copy of *fixS* in the CC1192 genome; therefore, either FixS is dispensable for N_2_ fixation in this symbiosis or an unidentified gene in CC1192 complements its function. Other M. ciceri strains, including M. ciceri WSM1271, WSM1284, and WSM1497, have a similar-sized *repABC*-type plasmid in their genomes (40, 50, 51); therefore, it is possible that these replicons may be widely conserved among this species.

The N_2_ fixation efficiency of both R7ANS and field-isolated exconjugants harboring ICE*Mc*Sym^1192^ was indistinguishable from that of CC1192 on C. arietinum. Importantly, ICE*Mc*Sym^1192^ was identified in all field isolates tested, and these 11 strains were distributed across three different clades, based on 16S rRNA and core gene phylogenies, indicating that this ICE can support CC1192 rates of N_2_ fixation in a comparatively wide range of *Mesorhizobium* genetic backgrounds. This is in stark contrast to results obtained previously with WSM1271 and WSM1497, in which field-isolated (18, 32) and *in vitro* R7ANS exconjugants of WSM1271 (28) were less effective than the inoculant strains at fixing N_2_ with B. pelecinus, with some field-isolated strains being completely ineffective (18). Therefore, acquisition of a symbiosis ICE from a *Mesorhizobium* strain can yield novel microsymbionts that are as effective as the inoculant strain at fixing N_2_ with host legumes.

Although the 11 *Mesorhizobium* strains isolated from field-cultivated C. arietinum showed symbiotic effectiveness equivalent to that of the inoculant strain CC1192, there is evidence that suboptimally effective strains nodulate C. arietinum in Australia. In their study of cultivated C. arietinum inoculated with CC1192, Elias and Herridge (36) isolated 570 strains from nodules of C. arietinum sampled across 26 farms in eastern Australia and found that 86% of strains were not CC1192. Importantly, while most novel strains analyzed were as effective as CC1192 on C. arietinum, 41% were suboptimal at fixing N_2_. Although the symbiosis ICE was not identified in those strains, the fact that uninoculated C. arietinum sown into fields in Australia without a history of inoculation fails to form effective N_2_-fixing nodules (7) is strong evidence for a lack of compatible preexisting soil organisms capable of fixing N_2_ with this legume. Therefore, it is highly likely that the novel C. arietinum-nodulating organisms reported by Elias and Herridge (36) are the result of transfer of the CC1192 ICE into recipient soil *Mesorhizobium* spp., as was shown to be the case with the 11 strains analyzed in this work.

The field-isolated recipients of ICE*Mc*Sym^1192^ were shown to form three separate clades, some of which may constitute new species of *Mesorhizobium*. Very little is known of the diversity of *Mesorhizobium* soil populations in Australia, with *Bradyrhizobium* spp. most frequently being identified from isolations made from nodules of native legumes (52–55). Only two studies reported the isolation of limited numbers of *Mesorhizobium* strains from some species of Australian native legumes (38, 39), and the 16S rRNA gene sequences of those strains group closely with some of the C. arietinum field-isolated strains analyzed in this study. It is possible that the novel C. arietinum strains analyzed in this study evolved from the transfer of ICE*Mc*Sym^1192^ from CC1192 into *Mesorhizobium* microsymbionts of Australian native legumes, converting them into C. arietinum symbionts. However, there is no evidence for non-ICE*Mc*Sym^1192^ symbiosis genes in the genomes of the field isolates, as might be expected if the ICE had integrated into the genome of a preexisting symbiont. This suggests that the recipients of ICE*Mc*Sym^1192^ might instead have been nonsymbiotic *Mesorhizobium* saprophytes existing as part of the soil microbiota. In fact, nonsymbiotic *Mesorhizobium* spp. have been isolated from the rhizosphere of L. corniculatus growing at two separate field sites in New Zealand (56). It is thus possible that populations of nonsymbiotic *Mesorhizobium* spp. similarly exist in Australian soils and can act as recipients for symbiosis ICE transfer from *Mesorhizobium* inoculant strains.

Why ICE transfer into different *Mesorhizobium* strains sometimes leads to ineffective or poorly effective N_2_-fixing microsymbionts, such as for B. pelecinus (32, 51, 57, 58), or effective N_2_-fixing rhizobia, as presented in this study, is not clear. However, the interaction between expression of ICE and chromosomally carried genes required for symbiosis is likely to play a significant role. The reports of large “naturalized” populations of *Sinorhizobium* and *Rhizobium* strains present in Australian soils (12, 15, 17) may similarly be the result of horizontal transfer of symbiosis plasmids from inoculant strains to native soil bacteria. Determining how these populations of rhizobia have developed is crucial for continuing to harness rhizobium-legume interactions in farming systems to maximize nitrogen inputs and for understanding the selective forces driving evolution of symbiotic N_2_ fixation.

## MATERIALS AND METHODS

### Strains, plasmids, and media.

Bacterial strains and plasmids used in this study are detailed in Table 2. *Mesorhizobium* spp. were cultured at 28°C on half-strength Lupin agar (½LA) (59) or tryptone yeast extract (61). For ICE transfer experiments, exconjugants were selected on rhizobium defined medium (RDM) supplemented with 15 mM glucose (G/RDM) (62). Escherichia coli was cultured at 37°C on lysogeny broth (63). Where appropriate, antibiotics were supplied in the medium at the following concentrations: for *Mesorhizobium*: neomycin, 250 µg ml^−1^ for exconjugant selection and 80 µg ml^−1^ for routine culturing; gentamicin, 40 µg ml^−1^; tetracycline, 1 µg ml^−1^; for E. coli: kanamycin, 40 µg ml^−1^; gentamicin, 10 µg ml^−1^; tetracycline, 10 µg ml^−1^. Medium for E. coli ST18 was also supplemented with 5-aminolevulinic acid at 50 µg ml^−1^.

**TABLE 2:** Strains and plasmids used in this study

Strain or plasmid	Genotype or alternative name^*a*^	Reference or source
Strains		
CC1192	Wild-type Mesorhizobium ciceri symbiovar ciceri CC1192, harboring ICE*Mc*Sym^1192^	7
R7A	Wild-type Mesorhizobium japonicum field isolate of ICMP 3153, harboring ICE*Ml*Sym^R7A^	30
R7ANS	Nonsymbiotic derivative of R7A; lacking ICE*Ml*Sym^R7A^ and harboring BHR vector pPR3; Nm^r^	26
MCC69	CC1192 derivative, cured of plasmid pMc1192	This study
MCC70	CC1192 derivative, cured of plasmid pMc1192	This study
MCC110	R7ANS exconjugant carrying ICE*Mc*Sym^1192^ integrated at Ser--tRNA	This study
MCC111	R7ANS exconjugant carrying ICE*Mc*Sym^1192^ integrated at Ser--tRNA	This study
WSM4303	Nat2 from field-cultivated C. arietinum; *Mesorhizobium* sp.	36
WSM4304	Nat3 from field-cultivated C. arietinum; *Mesorhizobium* sp.	36
WSM4305	Nat4 from field-cultivated C. arietinum; *Mesorhizobium* sp.	36
WSM4306	Nat5 from field-cultivated C. arietinum; *Mesorhizobium* sp.	36
WSM4307	Nat7 from field-cultivated C. arietinum; *Mesorhizobium* sp.	36
WSM4308	Nat8 from field-cultivated C. arietinum; *Mesorhizobium* sp.	36
WSM4310	Nat18 from field-cultivated C. arietinum; *Mesorhizobium* sp.	36
WSM4311	Nat19 from field-cultivated C. arietinum; *Mesorhizobium* sp.	36
WSM4312	Nat20 from field-cultivated C. arietinum; *Mesorhizobium* sp.	36
WSM4313	Nat21 from field-cultivated C. arietinum; *Mesorhizobium* sp.	36
WSM4315	Nat28 from field-cultivated C. arietinum; *Mesorhizobium* sp.	36
Rlv3841	Wild-type Rhizobium leguminosarum symbiovar viciae 3841	72
DH5α	Escherichia coli strain for cloning; F^−^ ϕ80*lacZ*ΔM15 Δ(*lacZYA-argF*)*U169 recA1 endA1 hsdR17*(r_K_^−^ m_K_*^+^*) *phoA supE44 thi-1 gyrA96 relA1*	Invitrogen
ST18	Escherichia coli; S17-1Δ*hemA thi pro hsdR* M^−^ with chromosomally integrated RP4-2Tc::Mu:Km^r^::Tn*7* Tra^+^ Tri^+^; Sm^r^	73
Plasmids		
pJET1.2/Blunt	PCR product cloning vector; Ap^r^	Thermo Fisher Scientific
pHP45-ΩSmSp	pHP derivative with ΩSmSp cassette; Sm^r^, Sp^r^	74
pJQ200SK	pACYC derivative; P15A origin of replication insertional mutagenesis inactivation vector; Gm^r^, Suc^s^	75
pRK2013	Helper plasmid used for mobilizing plasmids; ColE1 replicon with RK2 *tra* genes; Km^r^	76
pMCC6	pSacB carrying 5,274-bp *repABC* region from M. ciceri symbiovar WSM1271	This study
pSacB	BHR vector carrying inducible IPTG promoter and *sacB* gene; Nm^r^, Suc^s^	28
pPR3	BHR pPROBE-KT carrying *nptII* promoter from pFAJ1708; Nm^r^	77

a Nm^r^, neomycin resistance; BHR, broad host range; Km^r^, kanamycin resistance; Sm^r^, streptomycin resistance; Ap^r^, ampicillin resistance; Sp^r^, spectinomycin resistance; Gm^r^, gentamicin resistance; Suc^s^, sucrose sensitivity; IPTG, isopropyl-β-d-thiogalactopyranoside.

### Curing pMc1192 from CC1192.

Plasmid pMc1192 was cured from M. ciceri CC1192 using a plasmid incompatibility approach, by cloning the *repABC* region from pMESCI01 of M. ciceri sv. biserrulae WSM1271 into the suicide vector pSacB. RepABC in pMESCI01 shares 100% identity with RepABC in pMc1192. To construct the plasmid-curing vector pMCC6, a 5,274-bp region containing the *repABC* region of pMESCI01 (Mesci_6410-6412) was PCR amplified with primers 13 and 14, containing 5′ BamHI and XbaI tails, and the region was directionally cloned into pSacB, forming pMCC6. Plasmid pMCC6 was then transformed into ST18 and subsequently conjugated into CC1192 in a biparental mating performed in duplicate independently grown cultures of donor and recipient cells. Transconjugants were selected on G/RDM supplemented with neomycin with no added 5-aminolevulinic acid. Loss of plasmid pMc1192 was initially screened by PCR with primer pairs pr1 and pr2, pr3 and pr4, and pr5 and pr6, each designed to amplify approximately 500-bp amplicons in three equidistant regions around pMc1192. Strains that did not yield expected products, consistent with the loss of pMc1192, were cured of pMCC6 by plating on RDM medium supplemented with 5% (wt/vol) sucrose and counterselected for loss of neomycin resistance (carried on pMCC6), producing MCC69 and MCC70, each derived from independent matings of CC1192 and ST18 (pMCC6) cells. To visualize curing of pMc11192, MCC69 and MCC70 were subjected to Eckhardt gel electrophoresis using a modified version of the procedure described previously (64). Briefly, the two plasmid-cured derivatives, MCC69 and MCC70, wild-type CC1192, and the reference strain Rlv3841 were grown in triplicate to optical densities at 600 nm of approximately 0.3, a 200-μl aliquot of culture was then chilled on ice for 10 min before 1 ml of cold 0.3% (wt/vol) *N*-lauryl sarcosine in Tris-borate-EDTA (TBE) buffer was added and mixed by inversion, and the mixture was incubated on ice for an additional 10 min. The mixture was centrifuged (20,000 × *g* for 5 min at 4°C) and aspirated before gentle resuspension in 25 μl lysis solution (0.1 mg ml^−1^ lysozyme, 10% [wt/vol] sucrose, 10 µg ml^−1^ RNase A in 1× TBE). Immediately, 20 μl of the sample was loaded onto 0.75% (wt/vol) agarose with 1% (wt/vol) SDS and left for 30 min to settle before electrophoresis at 70 V for 16 h at 4°C and subsequent UV visualization after staining with ethidium bromide.

### *In vitro* ICE transfer experiments.

The ICE*Ml*Sym^R7A^-devoid R7A derivative strain R7ANS harbors the broad-host-range vector pPR3, encoding neomycin resistance, to facilitate selection against neomycin-sensitive ICE-donor strains. R7ANS was mated with CC1192 as described previously (28) except that neomycin was substituted for tetracycline in the selective medium. PCR screening was carried out on a total of 10 exconjugants selected from four independent mating experiments targeting the Ser--tRNA (Meslo_RS0233700) with a 16-bp sequence at its 3′ end identical to the core regions of ICE*Mc*Sym^1192^, using primer pairs pr10 and pr11 (binding across the putative *attL* junction of the R7ANS chromosome and ICE*Mc*Sym^1192^) and pr9 and pr12 (binding across the putative *attR* junction) (Table 3). Two of these exconjugants (MCC110 and MCC111) were selected for whole-genome sequencing.

**TABLE 3:** Primers used in this study

Primer no.	Name	Oligonucleotide sequence (5′ to 3′)	Replicon specificity or source
pr1	pMc1192-Fa	CGTTCGGACTTGAACCAGGA	pMc1192
pr2	pMc1192-Ra	CCTCAAAGCTGGCATCGAAC	pMc1192
pr3	pMc1192-Fb	GATCAATGGTGCGCGAGAAC	pMc1192
pr4	pMc1192-Rb	CGCTGTTTCGACGGTTTGTT	pMc1192
pr5	pMc1192-Fc	TTCCCCGAACGAGATTGCAA	pMc1192
pr6	pMc1192-Rc	AAGCGCGATCGACAGATGAT	pMc1192
pr7	CC1192_attB_F	GTTGTCGGGACTGTTGTTGG	CC1192 chromosome
pr8	CC1192_attB_R	TTGGTTTCTCCTCGAAGCGG	CC1192 chromosome
pr9	CC1192_attP_F	GCCGATTGTCACAGGCTACT	ICE*Mc*Sym^1192^
pr10	CC1192_attP_R	CGGACGAGATACCAGATGCC	ICE*Mc*Sym^1192^
pr11	R7ANS_attB_F	GTTATTGGCCGGCAAAGACC	R7ANS serine
pr12	R7ANS_attB_R	TTTCCGACCTACACGCTCAC	R7ANS serine
pr13	RepABC F BamHI	ATCAGGGATCCGTTGACCTCCGCATGCAAAC	This study
pr14	RepABC R XbaI	ATCAGTCTAGAGTCAATCTCACCAGGGCCAG	This study

### Sequencing, whole-genome assemblies, and alignments.

Sanger sequencing of PCR amplicons was performed by the Australian Genome Research Facility. For whole-genome sequencing, genomic DNA of plasmid-cured strain MCC69, R7ANS exconjugants MCC110 and MCC111, and field-isolated strains WSM403, WSM4304, WSM4305, WSM4306, WSM4307, WSM4308, WSM4310, WSM4311, WSM4312, WSM4313, and WSM4315 was extracted using a Qiagen blood and tissue DNeasy extraction kit (catalogue number 69054) according to the manufacturer’s instructions. Concentration and purity were analyzed with a NanoDrop One spectrophotometer (Thermo Fisher Scientific). Illumina MiSeq 2 × 250-bp paired-end reads (ACCESS Research, Murdoch University) were used to produce draft genomes of all strains. *De novo* genome assemblies were performed using SPAdes v3.10.1 software (65). Illumina sequencer adapter contamination was removed with neonsclip v0.132 (https://github.com/Victorian-Bioinformatics-Consortium/nesoni), and reads were corrected using Lighter v1.1.1 (66). Genomes were annotated using Prokka (67). NCBI accession numbers for assembled and annotated genomes can be found in Table S1 in the supplemental material. For whole-genome BLASTN comparisons, BLAST Ring Image Generator (BRIG) v0.9.5 (68) was used to produce BLASTN (options: -ungapped, -word_size 2000, upper and lower threshold 99%) alignments of sequence contigs or scaffolds of R7ANS exconjugant strains or field isolates with the complete genome of CC1192 (40).

Phylogenetic genome analysis was performed with bcgTree (69), which uses a concatenated alignment of 107 core genes conserved across bacterial genera to compare organisms. The tree was constructed using the RAxML (Randomized Axelerated Maximum Likelihood) program (70) with bootstraps set at 100. Phylogenetic and molecular evolutionary analyses of 16S rRNA gene sequences were performed using Geneious v11.1.5 to construct a neighbor-joining tree using the Tamura-Nei method to determine genetic distances with 5,000 replications. Included in the analysis were field-isolated C. arietinum-nodulating strains WSM4303, WSM4304, WSM4305, WSM4306, WSM4307, WSM4308, WSM4310, WSM4311, WSM4312, WSM4313, and WSM4315, along with CC1192, a selection of strains used as inoculants for other commercial legume species, *Mesorhizobium* type strains, and strains isolated from native Australian legume species. A list of NCBI gene or genome accession numbers for each strain can be found in Table S2.

### Assessment of N_2_ fixation with C. arietinum and statistical analysis.

Cicer arietinum cv. Neelam was grown in free-draining sterile sand in a glasshouse maintained at 22°C, as described by Yates et al. (71), where growth of legumes is limited by N deficiency except when they are nodulated by N_2_-fixing rhizobia. Briefly, seeds were surface sterilized in 70% ethanol (1 min), followed by 4% NaOCl (3 min), rinsed in six successive washes with sterile deionized water, and imbibed in the final wash for 5 min. Surface sterilized seeds were pregerminated on 0.9% (wt/vol) agar for several days at room temperature until emergence of the radicals and were sown aseptically into 1-liter pots (170 by 80 by 80 mm) containing a coarse sand mixture prepared as described by Yates et al. (71), adjusted to pH 6.5 with a 5 g/liter solution of Fe_2_(SO_4_)_3_ prior to steam sterilizing. Each pot was sown with two seedlings and thinned to one plant per pot upon emergence of shoots.

Cultures of rhizobia were incubated on ½LA plates (59) for 5 days at 28°C, and cells were suspended in 1% (wt/vol) sucrose at 10^8^ cells ml^−1^. C. arietinum treatment pots were either uninoculated (N-starved and N-fed controls) or inoculated separately with 1 ml of the cell suspension, while uninoculated pots received 1 ml of sterile deionized water. All pots were protected from airborne contamination initially by plastic cling film and then by sterile Alkathene beads as described by Yates et al. (71). Each treatment consisted of five replications, which were randomized and maintained with sterile water and CRS plant growth nutrient solution (71) as required. Uninoculated N-fed control treatments received 5 ml of 0.1 M KNO_3_ weekly.

Three separate glasshouse experiments were conducted to assess N_2_ fixation of strains with C. arietinum. The first experiment evaluated the N_2_ fixation effectiveness of 11 strains isolated from field-cultivated C. arietinum growing in the northern grain belt of eastern Australia in an earlier study by Elias and Herridge (36). These strains (WSM4303, WSM4304, WSM4305, WSM4306, WSM4307, WSM4308, WSM4310, WSM4311, WSM4312, WSM4313, and WSM4315) were shown previously to be different from the inoculant strain CC1192 by 16S rRNA gene sequencing and were isolated from paddock TA17 (near Moree), a site where C. arietinum had not been grown for 10 years. The second experiment measured the effectiveness of R7ANS exconjugant strains MCC110 and MCC111, harboring ICE*Mc*Sym^1192^, compared with R7A, R7ANS, and wild-type CC1192. The third experiment assessed the symbiotic phenotype of plasmid-cured derivatives of CC1192, compared with the wild-type strain.

To assess N_2_ fixation effectiveness of the 11 field isolates and the plasmid-cured derivatives of CC1192, plants were harvested at 49 or 44 days postinoculation, respectively, by carefully removing the roots from the soil and washing the root systems. Nodules were excised from the roots, and plant shoots were separated from the roots at the hypocotyl; both shoots and nodules were then dried at 60°C until they were desiccated prior to weighing. For the R7ANS exconjugant strains, assessment of nitrogenase activity was performed on intact plants prior to shoot and nodule biomass harvesting. Briefly, plants were harvested at 49 days postinoculation by removing them carefully from pots and soil substrate and transferring them to 1,000-ml Duran bottles with silicone septa. A total of 2% (vol/vol) acetylene was added to each bottle, and the rate of acetylene reduction was determined at 20°C as described by Yates and colleagues (71). Initial screening at time points of 1, 2, and 3 h indicated that the acetylene reduction relationship versus time was linear; thereafter, samples were extracted at 2 h. Following the acetylene reduction assay, shoots and roots were harvested as described above. The variance of the means of the dry foliage weights and nodule weights and the various acetylene reduction assay parameters was assessed by performing one-way analysis of variance (ANOVA), and the significant difference between the treatment means was analyzed by the Tukey honestly significant difference (HSD) *post hoc* test at an α value of 0.05 using IBM SPSS Statistics v24.

### Data availability.

Genome sequences generated in this study are available from NCBI under the following accession numbers: NZ_VFTE00000000 (WSM4303), NZ_NSFX00000000 (WSM4304), NZ_VFTD00000000 (WSM4305), NZ_VFTC00000000 (WSM4306), NZ_VFTB00000000 (WSM4307), NZ_NSFW00000000 (WSM4308), NZ_VFTA00000000 (WSM4310), NZ_NSFV00000000 (WSM4311), NZ_NSFU00000000 (WSM4312), NZ_NSFT00000000 (WSM4313), NZ_VFSZ00000000 (WSM4315), JADAMJ000000000 (MCC110), JADAMK000000000 (MCC111), and SRX9131521 (MCC70).

## Supplementary Material

Supplemental file 1

Supplemental file 2
