# Supplementary material for: Evolution of Diverse Effective N2-Fixing Microsymbionts of Cicer arietinum following Horizontal Transfer of the Mesorhizobium ciceri CC1192 Symbiosis Integrative and Conjugative Element
Source: Appl Environ Microbiol. 2021 Feb 12;87(5):e02558-20. doi: 10.1128/AEM.02558-20 (PMC8090884; doi:10.1128/AEM.02558-20)
Supplement: Supplemental file 2 [file AEM.02558-20-s0002.pdf]

**Supplemental Material**

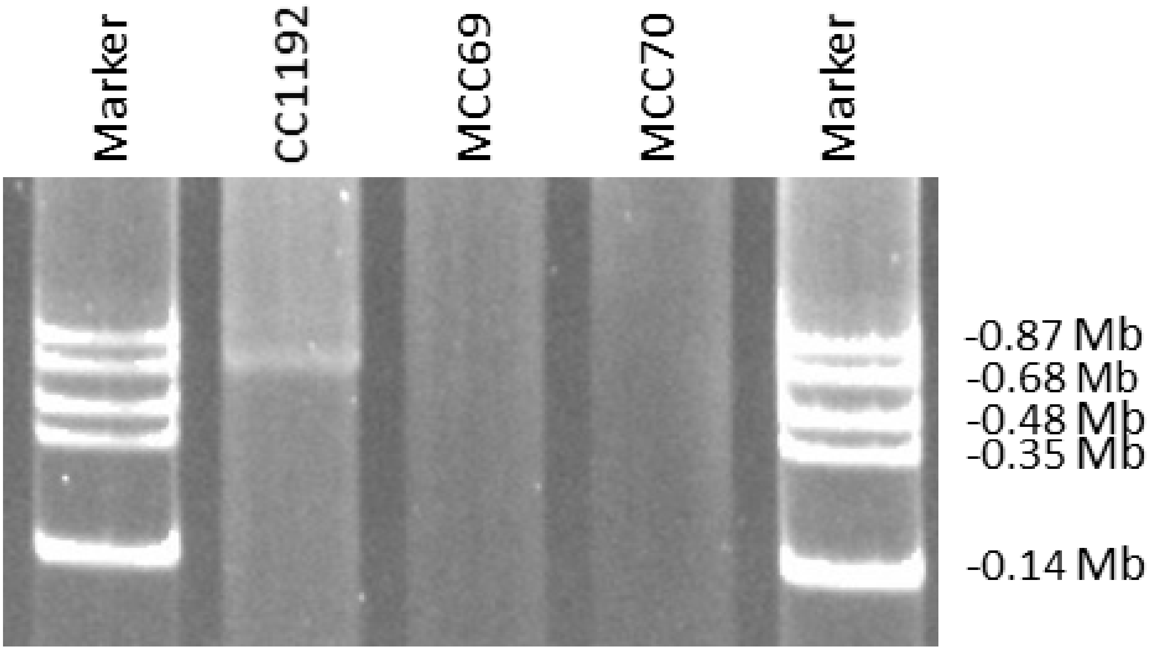


**Figure S1:** Eckhardt gel electrophoresis of wild-type CC1192 and two plasmid-cured derivatives, MCC69 and MCC70 at $OD_{600}$ of 0.497,0.376 and 0.359 respectively. The $OD_{600}$ of the marker strain Rlv3841) was 0.194, with bands corresponding to known plasmid replicons of pRL12 (0.87-Mb), pRL11 (0.68-Mb), pRL10 (0.48-Mb), pRL9 (0.35-Mb) and pRL8/pRL7 (0.14 and 0.15-Mb). A band corresponding to 0.65-Mb is present for CC1192 and absent in MCC69 and MCC70.

**Table S2:** GenBank whole genome sequence (WGS) or 16S rDNA accessions of *Mesorhizobium* strains selected for the construction of the genome tree and 16S rDNA gene phylogenetic tree. Alternative strain ID in parenthesis. *Where a WGS was available the 16S rDNA was extracted. **16S rDNA of *Mesorhizobium* sp. WSM2240 not available on NCBI database (pers.comm E. Watkin). *Rhizobium leguminosarum* bv. *viciae* USDA2370$^T$ included as an outgroup for the construction of the 16S rDNA tree

| **Strain ID** | **GenBank WGS accession** | **16S rDNA accession*** | **Host** |
|---|---|---|---|
| *M. alhagi* CCNWXJ12-2$^T$ | | EU169578 | *Alhagi sparsifolia* |
| *M. amorphae* ACCC 19665$^T$ | | AF041442 | *Amorpha fruticosa* |
| *M. australicum* WSM2073$^T$ | CP003358 | | *Biserrula pelecinus L.* |
| *M. caraganae* CCBAU 11299$^T$ | | EF149003 | *Caragana sp.* |
| *M. ciceri* ca181 | SAMN02470606 | | *Cicer arietinum* |
| *M. ciceri* CC1192 | CP015062 | | *Cicer arietinum* |
| *M. ciceri* CMG6 | AWZS00000000 | | *Cicer arietinum* |
| *M. ciceri* sv. *biserrulae* WSM1271$^T$ | NC_014923 | | *Biserrula pelecinus* L |
| *M. ciceri* sv. *biserrulae* WSM1284 | CP015064 | | *Biserrula pelecinus* L |
| *M. ciceri* sv. *biserrulae* WSM1497 | NZ_CP021071 | | *Biserrula pelecinus* L |
| *M. ciceri* UPM-Ca7$^T$ | | NR_025953 | *Cicer arietinum* |
| *M. ciceri* WSM1293 | KI911319 | | *Lotus sp.* |
| *M. erdmanii* USDA 3471$^T$ | AXAE00000000 | | *Lotus corniculatus* |
| *M. gobiense* CCBAU 83330$^T$ | | EF035064 | *Cicer arietinum* |
| *M. hawassense* AC99b$^T$ | | GQ847899 | *Sesbania sesban* |
| *M. huakuii* 7653R$^T$ | CP006581 | | *Astragalus sinicus* |
| *M. huakuii* CCBAU 2609 $^T$ | | D13431 | *Astragalus sinicus* |
| *M. japonicum* R7A | CP033366 | | *Lotus sp.* |
| *M. loti* LMG6125$^T$ (DSM 2626$^T$) | QGGH00000000 | | *Lotus corniculatus* |
| *M. japonicum* MAFF303099$^T$ | BA000012 | | *Lotus corniculatus* |
| *M. loti* SU343 | LYTL00000000 | | *Lotus sp.* |
| *M. mediterraneum* UPM-Ca36$^T$ | NPKI00000000 | | *Cicer arietinum* |
| *M. muleiense* CCBAU 83963$^T$ | FNEE00000000 | | *Cicer arietinum* |
| *M. oceanicum* B7$^T$ | CP018171 | | *Seawater* |
| *M. opportunistum* WSM2075$^T$ | NC_015675.1 | | *Biserrula pelecinus* L |
| *M. plurifarium* LMG 11892$^T$ (ORS1032T) | CCND00000000 | Y14158 | *Senegalia senegal* |
| *M. septentrionale* SDW 014$^T$ | | AF508207 | *Astragalus adsurgens* |
| *M. sophorae* ICMP 19535$^T$ | NNRI00000000 | | *Sophora microphylla* |

| | | | |
|---|---|---|---|
| *M. tarimense* CCBAU 83306$^T$ | | NR_044051 | *Lotus frondosus* |
| *M. temperatum* SDW 018$^T$ | NPKJ00000000 | | *Astragalus adsurgens* |
| *M. wenxiniae* WYCCWR 10195$^T$ | NPKH00000000 | | *Cicer arietinum* |
| *Mesorhizobium* sp. M1A | CP034455 | | *Cicer arietinum* |
| *Mesorhizobium* sp. M1B | CP034448 | | *Cicer arietinum* |
| *Mesorhizobium* sp. M2A | CP034446 | | *Cicer arietinum* |
| *Mesorhizobium* sp. M2C | RZTO01000000 | | *Cicer arietinum* |
| *Mesorhizobium* sp. M2D | RZOU01000000 | | *Cicer arietinum* |
| *Mesorhizobium* sp. M2E | SADP00000000 | | *Cicer arietinum* |
| *Mesorhizobium* sp. M3A | CP034451 | | *Cicer arietinum* |
| *Mesorhizobium* sp. M4B | CP034450 | | *Cicer arietinum* |
| *Mesorhizobium* sp. M5C | RZSY00000000 | | *Cicer arietinum* |
| *Mesorhizobium* sp. M6A | CP034452 | | *Cicer arietinum* |
| *Mesorhizobium* sp. M7D | CP034453 | | *Cicer arietinum* |
| *Mesorhizobium* sp. M8A | CP034454 | | *Cicer arietinum* |
| *Mesorhizobium* sp. M9A | CP034443 | | *Cicer arietinum* |
| *Mesorhizobium* sp. S | | Z94817 | *Goodia lotifolia* |
| *Mesorhizobium* sp. T | | Z94807 | *Acacia obliquinervia* |
| *Mesorhizobium* sp. T13 | | FJ19326 | *Acacia salicina* |
| *Mesorhizobium* sp. T14 | | FJ687988 | *Acacia stenophylla* |
| *Mesorhizobium* sp. T19 | | FJ867989 | *Acacia salicina* |
| *Mesorhizobium* sp. T20 | | FJ687990 | *Acacia stenophylla* |
| *Mesorhizobium* sp. U | | Z94819 | *Oxylobium ellipticum* |
| *Mesorhizobium* sp. WSM2240** | | | *Swainsona formosa* |
| *Mesorhizobium* sp. WSM4303 | NZ_VFTE00000000 | | *Cicer arietinum* |
| *Mesorhizobium* sp. WSM4304 | NZ_NSFX00000000 | | *Cicer arietinum* |
| *Mesorhizobium* sp. WSM4305 | NZ_VFTD00000000 | | *Cicer arietinum* |
| *Mesorhizobium* sp. WSM4306 | NZ_VFTC00000000 | | *Cicer arietinum* |
| *Mesorhizobium* sp. WSM4307 | NZ_VFTB00000000 | | *Cicer arietinum* |
| *Mesorhizobium* sp. WSM4308 | NZ_NSFW00000000 | | *Cicer arietinum* |
| *Mesorhizobium* sp. WSM4310 | NZ_VFTA00000000 | | *Cicer arietinum* |
| *Mesorhizobium* sp. WSM4311 | NZ_NSFV00000000 | | *Cicer arietinum* |
| *Mesorhizobium* sp. WSM4312 | NZ_NSFU00000000 | | *Cicer arietinum* |
| *Mesorhizobium* sp. WSM4313 | NZ_NSFT00000000 | | *Cicer arietinum* |
| *Mesorhizobium* sp. WSM4315 | NZ_VFSZ00000000 | | *Cicer arietinum* |
| *R. leguminosarum* bv. *viciae* USDA2370$^T$ | | JQ085246 | *Pisum* sp. |
